# Design and Effect of a Resin Infiltration Method to Enhance the Interlayer Adhesion of Additively Manufactured PEEK Parts

**DOI:** 10.3390/polym17212819

**Published:** 2025-10-23

**Authors:** Francesco Tamburrino, Beatrice Aruanno, Alessandro Paoli, Armando V. Razionale, Sandro Barone

**Affiliations:** Department of Civil and Industrial Engineering, University of Pisa, Largo Lucio Lazzarino, 1, 56126 Pisa, Italy

**Keywords:** Polyether ether ketone PEEK, Fused Filament Fabrication, resin infiltration, thermal post-processing, interlayer adhesion

## Abstract

This study investigates post-processing treatments aimed at enhancing the mechanical properties of Polyether Ether Ketone (PEEK) parts fabricated via Fused Filament Fabrication (FFF). FFF-printed PEEK components often exhibit anisotropy and weak interlayer adhesion, which limit their structural performance. To address these issues, a resin infiltration treatment is proposed that yields improvements in flexural strength and strength-to-weight ratio across specimens with different infill percentages. The effectiveness of resin infiltration is compared to that of a thermal post-processing treatment. Experimental results indicate that, although thermal treatment enhances crystallinity, it does not substantially improve interlayer bonding or mitigate anisotropy. In contrast, resin infiltration significantly enhances flexural strength, particularly in specimens with lower infill percentages, by effectively filling pores and reinforcing interlayer adhesion. Overall, the findings demonstrate that vacuum-assisted thermosetting resin infiltration is a promising post-processing technique for improving the mechanical performance of 3D-printed PEEK, achieving a mean flexural strength of up to 34 MPa, approximately 80% higher than that of untreated specimens with 100% infill. Additionally, a cost analysis comparing both post-processing methods is presented, highlighting the cost-effectiveness of resin infiltration as a viable solution to overcome the inherent limitations of FFF-printed PEEK.

## 1. Introduction and Background

Polyether ether ketone (PEEK) is a high-performance, semi-crystalline thermoplastic polymer suitable for applications requiring durability at high temperatures and in harsh environments [1]. Its molecular structure, with ether and ketone functional groups, provides high tensile strength (UTS ~110 MPa), stiffness (E ~4 GPa), and resistance to chemical degradation [2,3]. Additive Manufacturing (AM) has expanded the application range of PEEK, enabling the fabrication of complex geometries that are otherwise challenging to achieve with conventional manufacturing methods. This capability is particularly advantageous in fields such as biomedical engineering, where customized implants and devices are essential [4,5,6], as well as in aerospace and automotive industries, which demand lightweight components optimized through advanced topology design [7,8,9].

PEEK can be processed using several AM techniques, including Powder Bed Fusion (PBF)—specifically Selective Laser Sintering (SLS)—and Material Extrusion (MEX)—notably Fused Filament Fabrication (FFF) [2,10,11,12]. Its high melting temperature (~343 °C) and thermal stability make it well-suited for SLS, where a laser selectively fuses powdered material layer by layer. However, the use of powder-based processes requires stringent safety measures due to the potential hazards associated with powder handling [12].

FFF presents a more accessible and cost-effective alternative for processing PEEK, although it poses specific technical challenges. Compared to polymers such as PLA, PA, or ABS, PEEK requires much higher extrusion temperatures (380–420 °C), resulting in pronounced thermal gradients. These gradients hinder uniform cooling and limit the process window needed for optimal crystallinity and high mechanical properties [11]. The degree of crystallinity, which is influenced by thermal gradients during printing, is critical to the material’s mechanical performance: excessive crystallinity may result in brittleness, whereas insufficient crystallinity can lead to structural weakness [13,14]. Additionally, the printing process often suffers from limited control over interlayer bonding. Poor layer adhesion can compromise structural integrity, causing interlayer delamination and increasing the inherent anisotropic behavior of FFF-printed parts, which typically exhibit reduced mechanical strength along the *Z*-axis compared to the X-Y plane [15,16].

In this context, optimizing printing parameters is crucial for addressing challenges related to crystallinity, layer bonding, delamination, and anisotropy in FFF-printed PEEK components.

Previous research has mainly focused on refining thermal processing parameters, such as nozzle and bed temperatures, to control crystallinity. For instance, Jiang et al. [17] demonstrated that setting the nozzle temperature to 440 °C and optimizing printing speed can enhance the tensile strength of PEEK parts while effectively managing crystallinity. Similarly, Pu et al. [18] reported that controlling thermal cycles and employing a slow cooling rate during printing can stabilize crystallization, leading to improved mechanical properties, with a maximum crystal fraction of up to 50%.

Beyond controlling crystallinity, reducing delamination and anisotropy remains a further challenge. The interlayer bonding strength in the Z-direction is notably weaker than the in-plane strength, which can lead to delamination under mechanical stress [2,19]. This effect is exacerbated by the rapid cooling of extruded material, which induces thermal stresses [20]. Various strategies have been explored to address these limitations. For example, optimizing parameters such as extrusion temperature, layer thickness, and print speed has been shown to improve mechanical properties [21] and layer adhesion [22]. Specifically, higher nozzle temperatures can facilitate better filament melting, leading to improved layer adhesion and bonding [23].

While adjusting extrusion temperature, raster width, infill patterns, infill percentages, and extrusion speed can improve the chemical and mechanical properties of PEEK, challenges persist due to the inherent nature of the additive manufacturing process and the quality of feedstock filament. Porosity and limited interlayer adhesion remain critical issues in the fabrication of high-performance parts.

Given these challenges, post-processing treatments represent a promising avenue for further improving the mechanical properties of FFF-printed PEEK components. Indeed, this area offers significant potential for innovative research [24].

Thermal annealing is currently the most commonly adopted approach to enhance crystallinity and improve the mechanical properties of FFF-printed PEEK parts [25,26,27,28]. Although effective in increasing crystallinity, this treatment has limited impact on reducing porosity and enhancing interlayer bonding. For example, Lannunziata et al. [26] investigated the effects of both direct annealing during printing and post-printing annealing on the thermal and mechanical properties of FFF-printed PEEK. Both methods enhanced crystallinity, resulting in improved thermal stability, stiffness, and ultimate tensile strength. In [27], no significant improvements in mechanical properties were obtained by annealing at 200 °C and 300 °C, aside from a 14% increase in compression strength at slower printing speeds, likely due to improved interlayer adhesion. However, annealing did not mitigate the undesired porosity formed during 3D printing or alter the typical failure mechanism involving interlayer debonding.

In another study [28], thermal annealing at 200 °C resulted in a ~15% increase in tensile strength in 3D-printed PEEK, primarily attributed to increased crystallinity. Furthermore, a novel non-solvent vapor annealing method developed in [14] reduced the temperature required for the thermal treatment, enhancing strength, ductility, and fracture energy by 22.6%, 151.3%, and 109.1%, respectively. Nevertheless, the study focused on specimens printed in the optimal horizontal orientation, leaving the issue of interlayer bonding unaddressed.

Collectively, these studies demonstrate that while thermal annealing enhances crystallinity and improves the mechanical performance of 3D-printed PEEK, the inherent anisotropic behavior of FFF-printed components remains a critical limitation that warrants further investigation.

The study presented in this paper explores the design and effects of a novel post-processing technique based on vacuum-assisted thermosetting resin infiltration for FFF-printed PEEK. While thermal treatments have been extensively studied [25,26,27,29,30], thermosetting resin infiltration remains largely unaddressed in the literature, offering a promising route to enhance interlayer adhesion and reduce anisotropy in printed components. To date, only one study [31] has applied vacuum infiltration to specimens printed in a horizontal orientation, with the largest surface in contact with the build plate (XYZ, as defined by ISO 17295:2023 [32]). This configuration, however, does not account for anisotropic effects and represents the most mechanically favorable printing condition with respect to loading direction. To expand on this limited foundation, the present study focuses on evaluating the worst-case mechanical performance scenario. Specimens were therefore printed in a vertical orientation (ZXY, per ISO 17295:2023 [32]), with the smallest surface in contact with the build plate, to probe the inherent weakness of interlayer bonding in the Z-direction and the anisotropy typical of FFF-printed PEEK components. A comprehensive investigation was conducted using specimens with varying infill percentages (30% to 100%) to assess improvements in mechanical performance, with particular attention to the strength-to-weight ratio, an essential metric for high-performance applications involving advanced materials such as PEEK. Vacuum resin infiltration was compared to thermal annealing, with the as-printed condition serving as a baseline to isolate the effects of each post-processing treatment. Additionally, the cost implications of both techniques were evaluated to provide a holistic assessment of their industrial feasibility. To the best of our knowledge, this is the first systematic study investigating vacuum-assisted resin infiltration on vertically printed PEEK specimens, making this work a novel contribution to the field of additive manufacturing and post-processing of high-performance polymers.

## 2. Materials and Methods

### 2.1. Filament Characterization

In this study, Luvocom 3F 9581 PEEK filament (Prima SELECT™, Malmö, Sweden) with a diameter of 1.75 mm was used. Differential scanning calorimetry (DSC) was initially performed to better understand the material’s thermal properties and to obtain data relevant for thermal post-processing. DSC measures the heat flow into or out of a material during heating or cooling, providing information about thermal transitions such as melting, crystallization, and glass transition temperatures by detecting changes in heat capacity. A dynamic analysis was conducted by heating a 3.6 mg specimen from 60 °C to 400 °C at a constant rate of 10 °C/min to assess the material’s glass transition and melting temperatures.

### 2.2. Additive Manufacturing of PEEK

PEEK specimens were additively manufactured using CreatWare V7.0.2 slicing software and a CreatBot F430 3D printer (Zhengzhou City, Henan Province, China), equipped with two 0.4 mm direct-drive extruders. The system included a heated chamber (up to 70 °C), a build platform (up to 140 °C), and a nozzle capable of reaching 420 °C. Prismatic bars measuring 80 × 10 × 4 mm^3^ were fabricated for testing. Specimen dimensions and testing conditions followed ISO 178:2019 [33], the standard for evaluating the flexural properties of plastic materials. Specimens were printed in a vertical orientation (ZXY, according to the coordinate system in ISO 17295:2023 [32]), with their smallest surface in contact with the printer’s building plate.

The printing parameters (Table 1) were established based on preliminary experimental trials, insights from previous research [30], and the manufacturer’s recommendations for optimizing the filament printing. These parameters were applied consistently throughout specimen fabrication.

### 2.3. Design of Experimental Models

Materials fabricated using MEX exhibit anisotropy. Based on this, the study aimed to evaluate the worst-case mechanical performance scenario, specifically when a component is printed vertically and subjected to loading conditions that challenge interlayer adhesion.

Accordingly, the research examined the effects of varying infill percentages and post-processing treatments on the material’s flexural properties. Specimens with three different infill percentages (70%, 50%, and 30%) were evaluated, while dense PEEK (100% infill), fabricated exclusively with perimeters/walls to ensure printability, served as the reference material. These values were selected at regular intervals in accordance with previous studies [34,35]. Each specimen was weighed after 3D printing and again after post-processing to assess performance metrics such as strength-to-weight ratio and to estimate into material cost.

To enhance the mechanical behavior of additively manufactured PEEK parts, a novel resin infiltration method was developed and compared to a thermal annealing post-processing technique. Five specimens per infill percentage were tested under three conditions: as-printed, thermally treated, and infiltrated. Dense specimens were excluded from infiltration due to process infeasibility. Although minor micropores may exist even in 100% infill specimens due to printing defects, they are neither interconnected nor large enough to permit infiltration.

**Figure 1:**
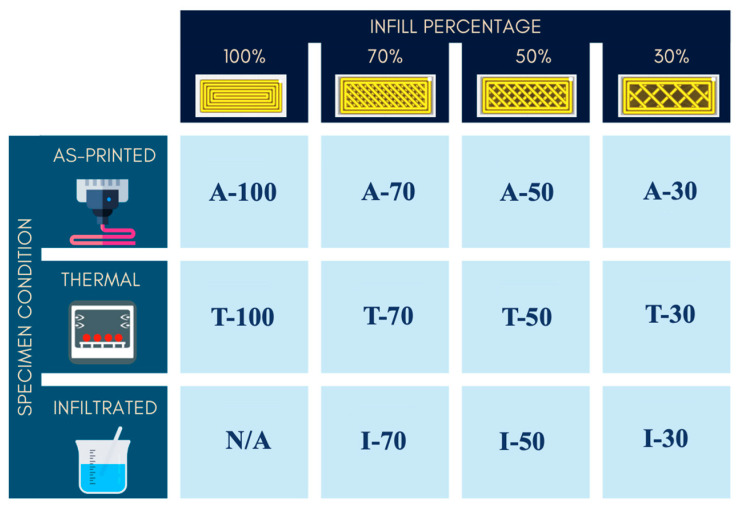
Schematic overview of the experimental design and specimen conditions tested.

Figure 1 illustrates the experimental design: 55 specimens, grouped in 11 sets, were tested. Each set was designated by a letter representing the specimen condition (“A” as-printed, “T” thermal treatment and “I” infiltration treatment), followed by the infill percentage.

### 2.4. Post-Processing of PEEK

#### 2.4.1. Resin Infiltration

A vacuum infiltration process was developed to enhance the properties of 3D-printed PEEK specimens. Infiltration was performed using a two-component thermoset system (Epoxy System HP-E120WSI), consisting of resin and hardener. This colorless resin is commonly used for laminating and vacuum infusion in composite materials, as well as for fabricating high heat-resistant components, due to its excellent dimensional stability and high glass transition temperature (125 °C).

The resin was selected for its low viscosity (~563 mPa·s), low density (1.11 g/cm^3^), high heat resistance, and its ability to undergo thermal polymerization at temperatures compatible with PEEK’s thermal resistance. Notably, its flexural strength is 110 MPa, nearly identical to that of PEEK. Other mechanical properties, such as elastic modulus (~3 GPa) and strain at break (~6%), are also comparable to those of 3D-printed PEEK.

Prior to infiltration, the resin–hardener system was mixed at the manufacturer’s recommended ratio (100 parts resin to 26 parts hardener by weight), using a magnetic stirrer to ensure uniform mixing. Vacuum infiltration was performed using a dedicated unit consisting of a chamber and pump. The maximum achievable vacuum level (−1 bar) was used to maximize the driving force for resin infiltration and reduce infiltration time, thereby enhancing process efficiency and repeatability. Preliminary trials involved weighing specimens before and after infiltration to estimate resin uptake and determine the minimum time required for maximum infiltration.

A specimen holder was used to ensure all specimens were vertically positioned and securely held inside a glass beaker during infiltration. Specimens were then submerged in the poured resin and infiltrated under vacuum for ten minutes, facilitating maximum pore infiltration and reducing overall porosity. Once the vacuum was released and the specimens removed, the resin remained trapped within the internal pores, as its viscosity and surface tension prevented it from flowing out. After infiltration, the outer surfaces of the specimens were manually cleaned with disposable tissues to remove excess resin. A subsequent six-hour heat treatment at 60 °C was performed to fully cure the infiltrated resin.

Finally, the infiltrated specimens were weighed to quantify changes in physical properties and to evaluate potential correlations with flexural performance.

**Figure 2:**
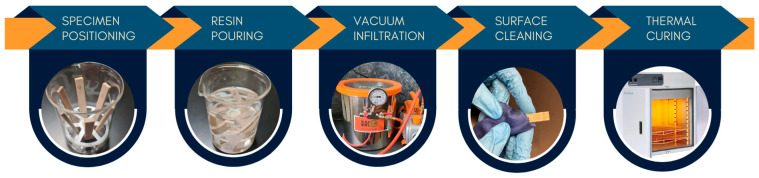
Schematic representation of the vacuum infiltration process.

A schematic representation of the vacuum infiltration process is shown in Figure 2.

**Figure 3:**
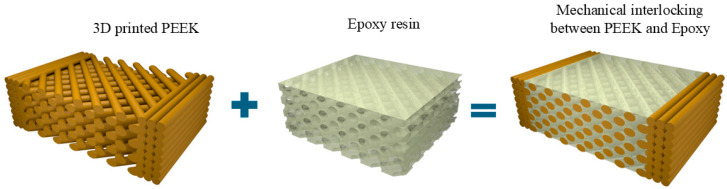
Schematic representation of the mechanical interlocking effect between PEEK and infiltrated epoxy resin.

The infiltration post-processing approach is not primarily aimed at promoting chemical bonding or adhesion at the PEEK–epoxy interface. Instead, it leverages the ability of the epoxy resin to infiltrate the porous structure of the 3D-printed PEEK, exploiting the interconnected voids present in parts printed at different infill percentages. The infiltration process aims to build a continuous phase and an interconnected network, establishing mechanical interlocking between the PEEK and epoxy. This strategy aims to enhance the interlayer strength of the post-processed PEEK through physical interconnection rather than chemical adhesion alone. Figure 3 illustrates this infiltration mechanism, highlighting how the epoxy penetrates the cavities created by the PEEK infill pattern, thereby improving structural cohesion through a mechanical interlocking effect.

To evaluate the infiltration efficiency, the percentage of void volume before and after infiltration was determined for I-30, I-50 and I-70 specimens by using the following equations:
Reference volume of dense PEEK specimen (infill percentage 100%), with:
$\rho_{PEEK}=1.31\;g/{cm}^{3}$
$V_{PEEK,100\%}=\frac{m_{PEEK,100\%}}{\rho_{PEEK}}$ (1)Volume of PEEK at different infill percentages (*x*%) with:
*x*% = 70%, 50%, 30%$V_{PEEK,x\%}=\frac{m_{PEEK,x\%}}{\rho_{PEEK}}$ (2)Mass of epoxy resin after infiltration$m_{resin,x\%}=m_{infiltrated,x\%}-m_{PEEK,x\%}$ (3)Volume of epoxy resin after infiltration, with: 
$\rho_{resin}=1.11\;g/{cm}^{3}$
$V_{resin,x\%}=\frac{m_{resin,x\%}}{\rho_{resin}}$ (4)Void fraction after printing with specimens at *x*% infill${∆V}_{void}^{print}\left(x\%\right)=\left(1-\frac{V_{PEEK,x\%}}{V_{PEEK,100\%}}\right)\times 100$ (5)Void fraction after infiltration with specimens at *x*% infill${∆V}_{void}^{infiltrated}\left(x\%\right)=\left(1-\frac{V_{PEEK,x\%}+V_{resin,x\%}}{V_{PEEK,100\%}}\right)\times 100$ (6)

The reference volume of the dense PEEK specimen (printed at 100% infill) was first determined using the average mass of the A-100 specimen set and the known density of PEEK, according to Equation (1). Then, with the same approach was measured the volume of specimens printed at different infill percentages (sets: I-70, I-50, I-30), with the mass measured before performing the infiltration process, using Equation (2). The mass of the infiltrated epoxy resin is given by (3), where $m_{PEEK,x\%}$ is the mass of the specimens at the *x*% infill condition before infiltration, and $m_{infiltrated,x\%}$ is the mass of the same specimens at the *x*% infill condition after the infiltration process. Consequently, the corresponding volume of the infiltrated Epoxy resin is (4). Finally, the initial void volume (i.e., void space due to partial infill of the AM process) and the post-infiltration residual voids were compared by computing the void fractions, respectively, with Equations (5) and (6). This approach allows a quantification of the internal volume percentage initially occupied by PEEK due to partial infill, the portion of the resulting void volume filled by resin, and the residual voids remaining after the infiltration process.

#### 2.4.2. Thermal Treatment

Besides as-printed conditions, a thermal post-processing method was developed and used as a comparison to validate the results obtained with the infiltration technique.

**Figure 4:**
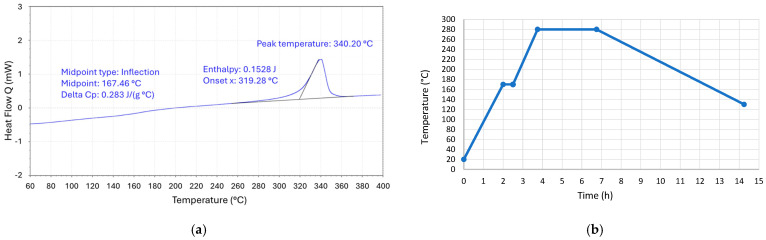
Differential Scanning Calorimetry of PEEK (**a**) and thermal cycle adopted for post-processing (**b**).

This treatment was based on a thermal cycle designed with reference to previous studies and primary data derived from the DSC analysis of the PEEK filament [26,27,30]. The DSC results (Figure 4a) indicated a glass transition temperature (*T_g_*) of approximately 167 °C and a melting temperature peak near 340 °C. Based on this information, the thermal cycle was developed as shown in Figure 4b.

Thermal annealing promotes the reorganization of polymer chains, enabling the crystallization of amorphous regions and enhancing the mechanical, thermal, and chemical resistance properties of semi-crystalline polymers. In this study, the process began with preheating the specimen to a temperature near PEEK’s glass transition (170 °C) and maintaining it for 0.5 h. The temperature was then gradually increased at a rate of 88 °C/h to 280 °C—well below the melting point—to prevent stress and deformation of the geometry. This temperature was maintained for 3 h. Subsequently, the material was cooled at a controlled, low cooling rate of 20 °C/h to 130 °C to minimize residual stress formation and ensure uniform crystallization, after which it was allowed to cool in an uncontrolled environment [36].

The entire thermal treatment lasted 14.25 h and was conducted using a Metal 3D Printing Starter furnace supplied by Sapphire 3D. The furnace, featuring a heated chamber measuring 152 × 152 × 159 mm^3^, could reach a maximum temperature of 1370 °C with a temperature control accuracy of ±1 °C. Specimens were packed and placed into a crucible filled with alumina powder during the treatment to ensure uniform heating and to protect the specimens from direct contact with the heat source during the thermal cycles.

### 2.5. Testing of PEEK

**Figure 5:**
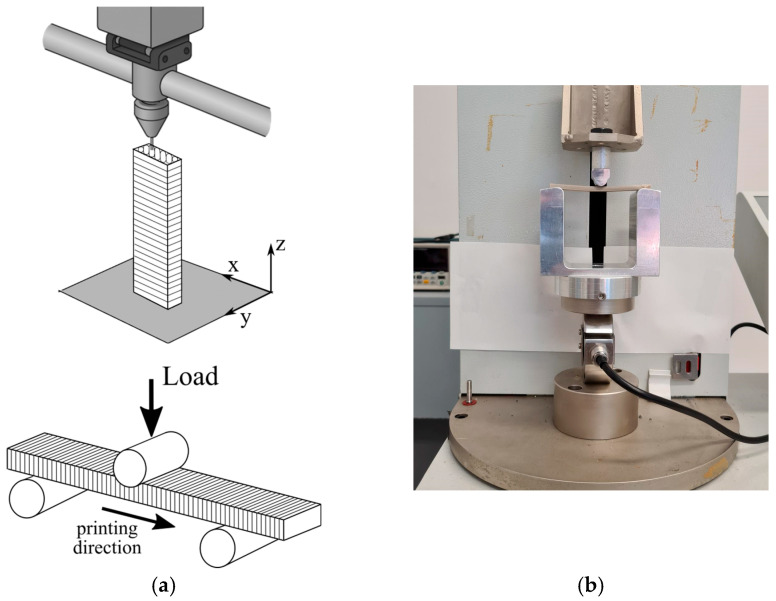
Schematic representation of the AM process and the three-point flexural test (**a**); actual flexural testing setup (**b**).

Three-point flexural tests were conducted at room temperature using an ESAT BPM testing machine equipped with a 0.5 kN load cell (Figure 5). Testing was carried out at a crosshead speed of 2 mm/min, with data acquisition at a frequency of 20 Hz. The resolution of the machine is ±0.01 N for force and ±0.01 mm for displacement.

The flexural strength of each sample was calculated in accordance with ISO 178:2019 [33], following the recommendations outlined in Section 9 of the Standard (Calculation and Expression of Results) and using the following equation:

(7) $$\sigma_{f}=\frac{3FL}{2bh^{2}}$$

where *F* represents the applied force, *L* is the span, and *b* and *h* correspond to the specimen’s width and thickness, respectively.

To analyze differences among the eleven experimental sets (Figure 1) and assess the effects of the independent factors and their interaction on the ultimate flexural strength, the following statistical analysis was carried out.

A priori power analysis was conducted using G*Power (v3.1.9.7) [37] to estimate the required sample size. Assuming a very large effect size (Cohen’s *f* = 0.60 [38,39]), *α* = 0.05, and power = 0.80, the minimum number of specimens required was calculated, and considering both cases of main effects and interaction, it was rounded to five per group. This is also consistent with ISO 178:2019 [33] and related material testing standards, which typically prescribe five replicates per condition.

The collected data were first tested for normality using the Shapiro–Wilk test. A two-way analysis of variance (ANOVA) was then performed to assess the effects of the two factors and their interaction. A full factorial design based on two independent factors, namely infill percentage (70%, 50%, and 30%) and specimen condition (as-printed, thermally treated, and infiltrated), was carried out.

In addition, effect sizes were computed using eta squared (*η*^2^) for each factor and the interaction. These values were then converted into Cohen’s *f* to perform post hoc power analysis using the following formula:

(8) $$f=\sqrt{\frac{\eta^{2}}{\left(1-\eta^{2}\right)}}$$

where significant effects were observed, pairwise comparisons were conducted using Tukey’s Honest Significant Difference (HSD) post hoc test. Tukey’s test adjusts for multiple comparisons, providing adjusted *p*-values (p-adj) for each pair of conditions. For each pairwise comparison, Cohen’s *d* was computed to estimate the effect size, using the difference in group means divided by the pooled standard deviation of the two groups.

Data from the as-printed and thermally treated dense specimens (sets A-100, T-100) were used as reference values and were excluded from the statistical analysis.

## 3. Experimental Results

**Figure 6:**
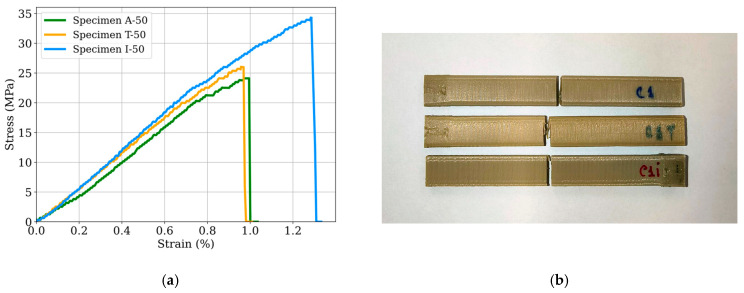
Representative stress–strain curves of 50% infill samples in the three different tested conditions (**a**) and corresponding fractured specimens (**b**).

Figure 6a displays representative stress–strain curves, highlighting the brittle fracture behavior consistently observed across all tested specimens. The plotted curves correspond to samples printed with 50% infill under three conditions: as-printed, thermally treated, and resin-infiltrated. Despite variations in flexural strength depending on the post-processing method, all specimens exhibit a sharp stress drop after the peak load, indicative of brittle failure. This fracture behavior is further confirmed by the corresponding fracture surface images shown in Figure 6b.

**Figure 7:**
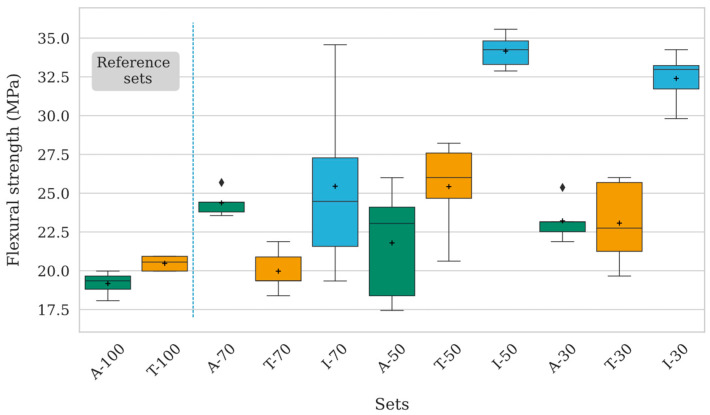
Box plot showing the flexural strength achieved for each specimen set: green (as-printed), orange (thermally treated), and cyan (infiltrated).

Figure 7 presents box plots of the flexural strengths for each set of specimens, as classified in Figure 1. The measured flexural strength ranges from approximately 17.5 MPa to 35.6 MPa. The specimens with the lowest flexural strength are those with 100% infill, in both as-printed and thermally treated conditions. Despite their lower strength, these two conditions show limited variability, indicating good repeatability and reliability, with the mean strength of A-100 being approximately 19 MPa. Conversely, infiltrated PEEK specimens generally exhibit the highest mean flexural strength, particularly I-30 and I-50, which reach 32 MPa and 34 MPa, respectively.

Although I-70 shows a higher mean flexural strength compared to as-printed and thermally treated conditions, it also presents greater variability among specimens. This deviation may be attributed to challenges encountered during the infiltration process, as the lower porosity of the 70% infill structure hinders effective resin penetration compared to 30% and 50% infill specimens. This issue is further evidenced by the inconsistent weight increase observed after infiltration, ranging from 0.27% to 2.49% (Table 2).

Overall, thermal treatment does not significantly enhance flexural strength compared to the as-printed condition, as indicated by the overlapping boxplots. As-printed specimens maintain relatively low but consistent flexural strength across different infill percentages, with a slight increase at 70% infill, while the lowest values are observed for the fully dense condition.

A two-way ANOVA was performed at a significance alpha level of 0.05 to evaluate the influence of infill percentage and post-processing condition on flexural strength. The results are as follows:

- Infill percentage (*F*(2) = 6.41, *p* < 0.005, *η*^2^ = 0.088, *f* = 0.312, *Power* = 0.418): the analysis shows a significant effect of infill percentage on flexural strength, indicating that variations in infill levels (30%, 50%, 70%) lead to statistically significant differences in strength;
- Specimen condition (*F*(2) = 36.82, *p* < 0.001, *η*^2^ = 0.508, *f* = 1.017, *Power* = 0.999): the different post-processing conditions (A, T, I) have a strong and significant effect on flexural strength, as highlighted also by the very large effect value (*f* ≫ 0.4);
- Interaction effect (*F*(4) = 5.61, *p* < 0.005, *η*^2^ = 0.155, *f* = 0.428, *Power* = 0.556): a statistically significant interaction between infill percentage and post-processing treatment was observed. This suggests that the impact of one variable (e.g., infill percentage) on flexural strength depends on the level of the other variable (e.g., post-processing treatment). For example, decreasing the infill percentage affects flexural strength differently in infiltrated specimens compared to as-printed or thermally treated specimens.

The Shapiro–Wilk test for normality of residuals confirmed that the residuals are normally distributed, thus satisfying the assumption of normality (*W* = 0.95, *p* > 0.05).

**Figure 8:**
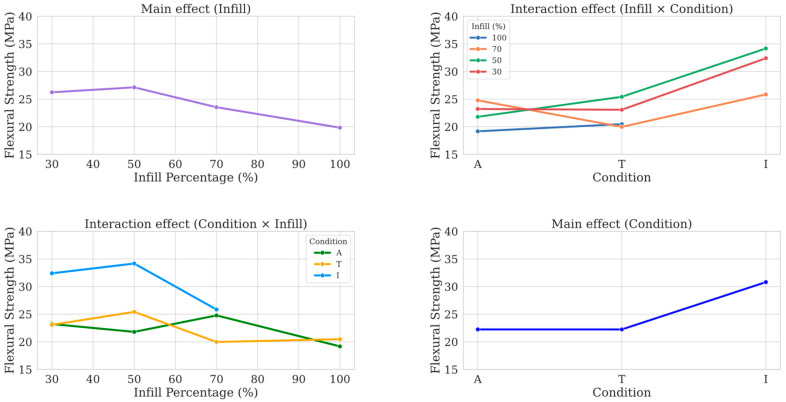
Main and interaction effects of infill percentage and post-processing treatments on ultimate flexural strength.

**Figure 9:**
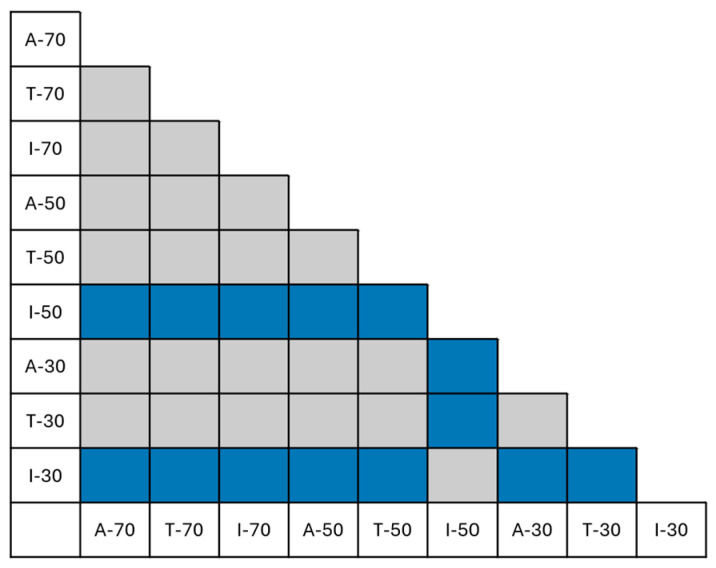
Tukey’s HSD matrix with blue cells for statistically significant pairs and gray cells for non-significant pairs.

Figure 8 illustrates the main and interaction effects of infill percentage and post-processing treatment on the ultimate flexural strength. Figure 9 presents the Tukey’s HSD results, identifying statistically significant differences between specific pairs of sets (*p* < 0.05, *reject* = *True*): blue cells indicate pairs that are significantly different, while gray cells represent pairs that are not. Notably, comparisons involving the infiltrated samples consistently showed large to extremely large effect sizes, as quantified by Cohen’s *d* (e.g., *d* > 5 in some cases), indicating very strong differences in flexural strength across post-processing methods. These values greatly exceed the typical threshold for a large effect (*d* > 0.8) [38] and suggest that the treatment produces not only statistically significant differences, but also meaningful improvements in performance.

The results for A-100 and T-100 are not statistically distinguishable and can be used as reference indicators for the other set conditions. As-printed and thermally treated specimens exhibit similar trends at equivalent infill percentages, whereas infiltrated specimens (I-30, I-50, I-70) consistently show higher flexural strength values, particularly at lower infill percentages (50% and 30%). Notably, I-50 significantly outperforms both T-50 and A-50, highlighting the effectiveness of infiltration in enhancing mechanical properties when material density is reduced.

PEEK is widely used in lightweight applications, where the strength-to-weight ratio is a key performance criterion. This study investigates the relationship between strength-to-weight ratio and the effects of infiltration on specimen performance.

As expected, specimens with 100% infill exhibited the highest weight, averaging 3.82 g, while as-printed specimens with 30% infill had the lowest weight, averaging 2.7 g. After infiltration, specimens with 70%, 50%, and 30% infill exhibited a mean weight of approximately 3.5 g.

Table 2 summarizes the mean weight, weight increment (%) after infiltration, and strength-to-weight ratio for all specimen types. The best-performing specimens in terms of strength-to-weight ratio were I-50 and I-30, confirming that infiltration significantly enhances flexural strength despite a moderate increase in weight.

**Figure 10:**
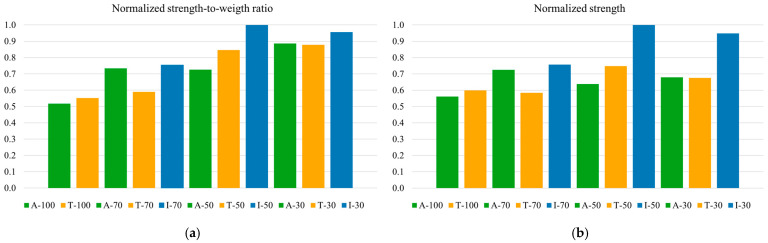
Normalized comparisons of strength-to-weight ratio (**a**) and flexural strength (**b**).

For comparison, both strength-to-weight ratio and flexural strength were normalized to their maximum values and are shown in Figure 10a,b. The performance gap between the A-100 and T-100 reference specimens and the best-performing infiltrated specimens increases. Furthermore, these figures reveal that while the I-50 and I-30 specimens demonstrate superior overall performance, the A-30 and T-30 specimens achieve notable strength-to-weight ratios due to their lower weight, with less pronounced differences compared to their infiltrated counterparts. This suggests that while infiltration significantly enhances absolute performance (strength), non-infiltrated specimens with 30% infill provide a competitive alternative when lightweight performance (strength-to-weight ratio) is prioritized.

**Figure 11:**
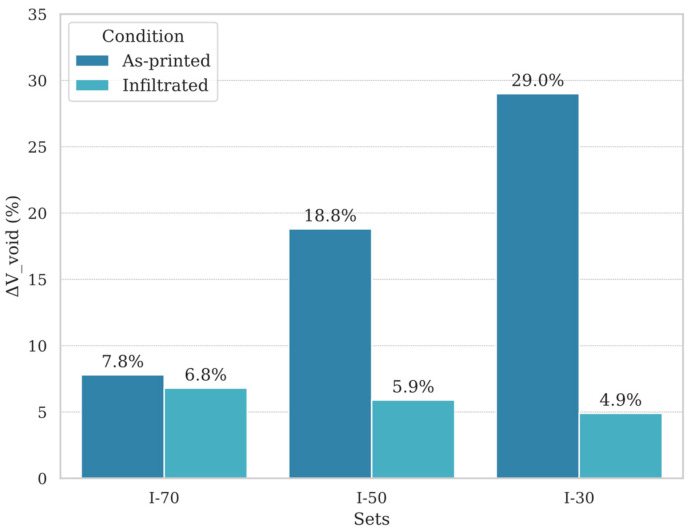
Void volume percentages for specimens before and after the infiltration process. The A-100 specimen was used as reference for the value $V_{PEEK,100\%}$ in Equations (5) and (6).

Figure 11 presents data on infiltration efficiency and residual void volume for specimens I-70, I-50, and I-30. The A-100 specimen, printed at 100% infill, was used as a reference, representing the maximum attainable density. For comparison, it was considered to have negligible or zero internal voids, although minor porosities may still exist even under full infill conditions. As expected, the post-print void volume (estimated with Equation (5)) increased with decreasing infill, with values of approximately 7.8%, 18.8%, and 29.0% for I-70, I-50, and I-30, respectively. After the infiltration process, a substantial reduction in void volume was observed across all cases. The remaining void volume (computed with Equation (6)) dropped to 6.8%, 5.9%, and 4.9% for I-70, I-50, and I-30, respectively. These results demonstrate that resin infiltration is more effective at lower infill levels, likely due to larger and more interconnected pores.

**Figure 12:**
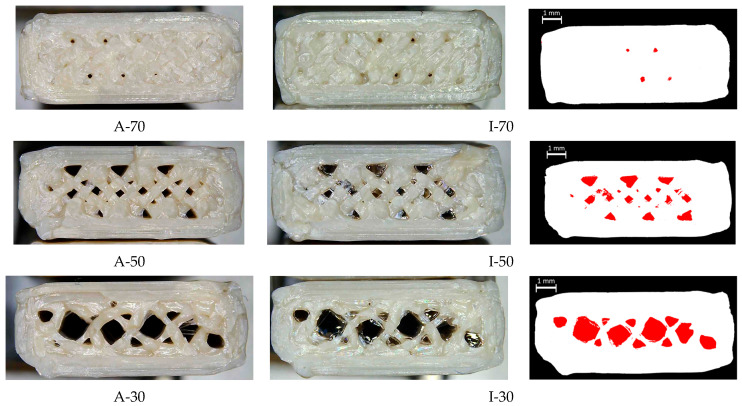
Cross-sectional observations of 70%, 50%, and 30% specimens in their as-printed (left column) and infiltrated (central and right column) conditions. Red areas in the right column evidence infiltrated epoxy resin.

The effectiveness of the infiltration process was further evaluated through digital microscopy of the cross-sections of fractured specimens, taken at the mid-span region to provide a representative view of the internal structure. Figure 12 presents a comparative view organized in three columns: the left column shows as-printed specimens with different infill percentage (A-70, A-50, A-30), revealing the characteristic initial porous structure; the middle column displays infiltrated specimens with the same infill percentage (I-70, I-50, I-30), where shiny, reflective areas indicate the presence of infiltrated epoxy resin. To enhance the visual distinction between the two phases, the right column shows the same images from the middle column after binarization, performed using a thresholding algorithm in MATLAB R2025b. In these processed images, red regions represent the infiltrated epoxy resin.

A clear difference in infiltration behavior was observed among the samples. In specimens I-50 and I-30, the resin was homogeneously distributed and visibly penetrated the internal structure. In contrast, specimen I-70 showed more limited infiltration, likely due to smaller pore size and lower pore connectivity, which hindered the resin flow and resulted in unfilled voids.

**Figure 13:**
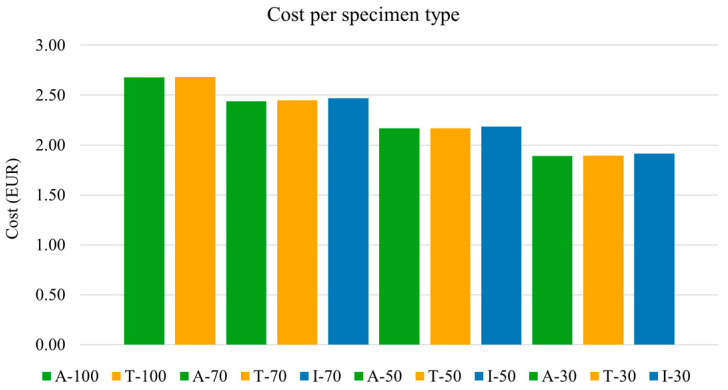
Material cost for each specimen set, expressed in EUR. Costs were calculated from the weight of each material component and its unit price.

To provide a comprehensive evaluation, material costs were also analyzed. Figure 13 presents the cost per specimen type, considering only raw material costs (excluding energy and processing costs). The raw materials used in this study included PEEK filament (current cost 700 €/kg) and a two-component thermoset epoxy system (current cost 20 €/kg). As expected, specimens with 100% infill incurred the highest cost, approximately 2.68 € per specimen, while specimens with 30% infill were the most cost-effective, at approximately 1.90 € per specimen. The additional cost due to infiltration treatment in specimens with 70%, 50%, and 30% infill was minimal (ranging from 0.01 to 0.03 € per specimen), resulting in negligible cost variations.

## 4. Discussion

Although the infiltration treatment of FFF PEEK specimens has been only marginally addressed in the literature, a comparison with previous studies on related topics effectively highlights the mechanical benefits of the proposed technique.

The study presented in [31] examined PEEK infiltration with a thermosetting resin; however, flexural tests were conducted with specimens loaded perpendicular to the printed layers, resulting in a mean strength of about 94 MPa. In contrast, in the present study, specimens were loaded parallel to the printed layers, under the most unfavorable conditions regarding anisotropy, thereby directly evaluating interlayer adhesion.

In [29], interlayer bonding after thermal annealing post-process was investigated through flexural testing. The findings indicated that heat treatments following 3D printing resulted in minimal improvements in interlayer bonding strength, suggesting that adhesion is predominantly determined during the printing process. While heat treatment increased crystallinity, the results demonstrated that this could be detrimental for vertically printed PEEK specimens. Higher crystallinity appears to hinder polymer chain diffusion across interfaces during annealing, negatively impacting interlayer bonding. The flexural strength values for these specimens ranged from approximately 6 MPa to 30 MPa, with a mean value of around 15 MPa.

In [40], 3D-printed PEEK reinforced with 10 wt% short carbon fibers was examined with microwave post-processing at different irradiation powers. An increase in tensile strength was observed, even in vertically printed specimens; however, the maximum tensile strength remained below 15 MPa.

The research presented in [26] partially overlaps with the present study, as it examined the effects of annealing and infill percentage on 3D-printed PEEK specimens. However, the focus was on horizontally printed specimens and mechanical performances in relation to weight. Their findings showed that while absolute mechanical performance generally improved with higher infill percentages, the best strength-to-weight trade-off occurred at 70% infill. Their study highlighted that partially dense specimens can be a viable option, especially when material is used under the most favorable conditions regarding anisotropy and interlayer bonding strength.

Further insights can be drawn from [41], where the authors investigated interlayer bonding using a pellet-based extrusion process. The highest interlayer shear strength obtained was 17.7 ± 0.6 MPa for high-viscosity PEEK grades processed at 420 °C. This value still remains significantly lower than those achieved in the present work. Similarly, in [42], the interlayer strength of the as-printed PEEK specimens reached only 13.4 ± 1.3 MPa, confirming the inherently weak bonding between layers in the Z-direction. Blending PEEK with amorphous analogs such as DPAPEEK or AFPEEK increased the interlayer strength up to approximately 23–24 MPa, yet the improvement remained moderate. This result underlines that even with chemical modifications of the polymer matrix, the interlayer adhesion of printed PEEK remains limited and well below the levels achieved through the infiltration treatment proposed.

The results of the present study provide valuable insights into the flexural strength, strength-to-weight ratio, and cost-efficiency of 3D-printed PEEK specimens with varying infill percentages and post-processing treatments.

The analysis of the flexural strength reveals distinct performance trends across the as-printed, thermally treated, and infiltrated specimens, indicating that both infill percentage and treatment type significantly influence mechanical behavior. The lowest flexural strength values were observed in specimens with 100% infill, both in the as-printed (A-100) and thermally treated (T-100) conditions. Despite having the highest material density, these specimens did not demonstrate enhanced strength; instead, their properties declined. This finding suggests that increasing infill percentage alone does not lead to proportional improvements in mechanical performance. This phenomenon might be explained by differences in deposition patterns between fully dense PEEK specimens and partially dense ones (e.g., with 70%, 50%, and 30% infill percentages). In fully dense specimens, concentric deposition patterns dominate, whereas in partially dense specimens, deposition paths intersect at different angles and may distribute loads more effectively across multiple directions. This could potentially reduce the directionality of the mechanical properties and enhance mechanical performance [43,44]. Conversely, infiltrated specimens, particularly those with lower infill percentages (I-30 and I-50), exhibited the highest flexural strengths, reaching mean values up to 34 MPa. This result represents an 80% increase over A-100 under perpendicular loading, highlighting the effectiveness of the infiltration process in enhancing interlayer strength of the structure, thus improving load-bearing capabilities. However, the significant variability in the flexural strength of I-70 specimens indicates that the infiltration process may be less effective at higher infill percentages, likely due to reduced interconnected porosity and difficulty in fully penetrating the material. The inconsistent weight increments observed (0.27–2.49%) suggest that achieving effective infiltration in denser specimens is challenging and may require further process refinement. In contrast, resin infiltration appears particularly effective in specimens with lower infill, where a larger proportion of the available void space is successfully filled. Notably, the most significant relative reduction was observed in specimen I-30, suggesting that larger and more interconnected pores may facilitate more efficient epoxy resin infiltration (Figure 11).

The two-way ANOVA confirmed that both infill percentage and treatment condition significantly affect flexural strength, with a notable interaction effect between these factors. This interaction implies that the influence of infill percentage on flexural strength is not uniform across conditions; for instance, infiltration has a more pronounced effect at lower infill levels.

Tukey’s HSD analysis further highlighted that specimens treated with infiltration (I-30 and I-50) significantly outperformed their as-printed and thermally treated counterparts, underscoring the effectiveness of infiltration in low-density applications. Notably, thermal treatment did not yield significant improvements in flexural strength across any set of specimens, suggesting that its effect on interlayer adhesion is minimal regardless of infill percentage.

In terms of the strength-to-weight ratio, as expected, the specimens with 100% infill had the highest weight and did not excel in strength-to-weight performance. In contrast, infiltrated specimens at 50% and 30% infill (I-50 and I-30) achieved optimal performance by maintaining high flexural strength while minimizing weight (about 8% less than 100% infill specimens). These results indicate that infiltration provides a viable method to enhance the absolute performance of low-density parts, achieving competitive strength-to-weight ratios and reinforcing the potential of this treatment for applications where lightweight performance is prioritized.

Interestingly, the A-30 set also demonstrated a notable strength-to-weight ratio due to its lower weight. These findings suggest that, while infiltration maximizes strength, non-infiltrated specimens with low infill percentages (30%) may serve as cost-effective alternatives where weight is prioritized over absolute strength. This balance between performance and material efficiency may be particularly advantageous in cost-sensitive applications.

Regarding cost analysis, as-printed specimens with 100% infill had the highest material cost (approximately 2.68 € per specimen), while those with 30% infill were the least expensive (1.90 € per specimen). The minimal cost increase associated with infiltration (ranging from 0.01 € to 0.03 € across infill percentages) demonstrates that the benefits of infiltration can be achieved with negligible impact on raw material costs. Moreover, although this study does not quantify energy costs, it is important to highlight that infiltration treatment, compared to the conventional thermal treatment, requires shorter processing cycles (6 instead of 14 h) and significantly lower temperatures (about five times lower), which would further amplify the cost difference. Consequently, infiltration emerges as a cost-effective means to significantly enhance flexural strength, particularly for specimens with lower infill percentages.

## 5. Conclusions

This study demonstrates that vacuum-assisted thermosetting resin infiltration is an effective post-processing strategy for enhancing the mechanical performance of vertically 3D-printed PEEK parts.

Across 30÷70% infill, infiltration increased flexural strength and strength-to-weight ratio with respect to as-printed and thermally treated conditions. The highest flexural strength reached 34 MPa at 50% infill, almost two times higher than the dense as-printed reference (~19 MPa), indicating improved interlayer adhesion and contributing to a reduction in anisotropy. The benefit diminished at 70% infill, consistent with limited resin uptake in denser structures.

Thermal annealing produced negligible changes in flexural strength at all infill levels, confirming that crystallinity gains did not translate into stronger interlayer adhesion. Also, as-printed specimens showed notably inferior mechanical performance with respect to the corresponding infiltrated sets.

Infiltration added only a minimal material-cost increase (~0.01÷0.03 € per specimen) while delivering higher absolute strength and competitive strength-to-weight ratios. The 50% and 30% infiltrated sets achieved mean values of ~9.70 MPa/g and ~9.27 MPa/g, respectively, substantially exceeding the ~5 MPa/g measured for the dense reference, at weights about 8% lower than the dense specimens. These attributes suit strength-critical yet weight-sensitive applications.

Overall, resin infiltration emerges as a practical, cost-efficient post-processing approach for 3D-printed PEEK. Future work should focus on improving infiltration in higher-density structures (e.g., tailored pore architectures and alternative resin systems), assess long-term durability under service conditions, and explore combinations with print-parameter optimization or complementary post-processing treatments.

## Figures and Tables

**Table 1:** Main process parameters set for PEEK manufacturing.

Parameter	Value
Extrusion temperature (°C)	418
Bed temperature (°C)	140
Chamber temperature (°C)	70
Printing speed (mm/s)	20
Layer height (mm)	0.15
Extrusion width (mm)	0.4
Infill pattern	Rectilinear
Infill angle (°)	±45
Bottom layers	3
Top layers	0

**Table 2:** Mean weight, weight increment range after infiltration and strength-to-weight ratios for all specimen types.

Set	Mean Weight (g)	St. Dev. (g)	Weight Increment Range (%) Post-Infiltration	Mean Strength to Weight Ratio (MPa/g)	St. Dev (MPa/g)
A-100	3.82	0.03	-	5.01	0.18
T-100	3.83	0.02	-	5.35	0.10
A-70	3.48	0.03	-	7.11	0.27
T-70	3.50	0.05	-	5.72	0.47
I-70	3.56	0.04	0.27 ÷ 2.49	7.31	1.60
A-50	3.10	0.01	-	7.03	1.19
T-50	3.10	0.01	-	8.20	0.97
I-50	3.56	0.02	13.61 ÷ 15.6	9.70	0.27
A-30	2.70	0.02	-	8.60	0.55
T-30	2.71	0.01	-	8.52	1.03
I-30	3.50	0.01	28.2 ÷ 30	9.27	0.49

## Data Availability

The original contributions presented in this study are included in the article. Further inquiries can be directed to the corresponding author.
